# Supplementary material for: Differential microstructural development within sensorimotor cortical regions: A diffusion MRI study in preterm and full-term infants
Source: Dev Cogn Neurosci. 2025 Aug 30;75:101610. doi: 10.1016/j.dcn.2025.101610 (PMC12446505; doi:10.1016/j.dcn.2025.101610)
Supplement: Supplementary file 1 — Supplementary material [file mmc1.docx]

# Supplementary Materials

**
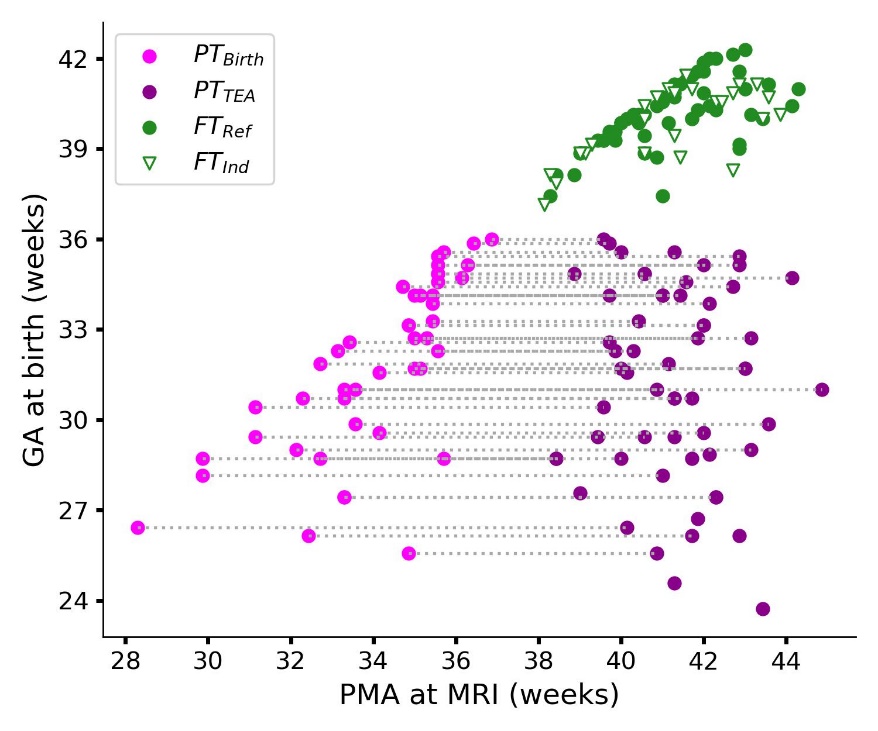
**

**Supp. Figure 1.** Distribution of post-menstrual age (PMA) at MRI and gestational age (GA) at birth for the 4 subject groups. Grey dotted lines connect longitudinal scans for the same subject. *Legend*: PT_Birth_: preterm group scanned close to birth, PT_TEA_: preterm group scanned at term equivalent age (TEA), FT_Ref_: reference full-term group, FT_Ind_: independent full-term group.


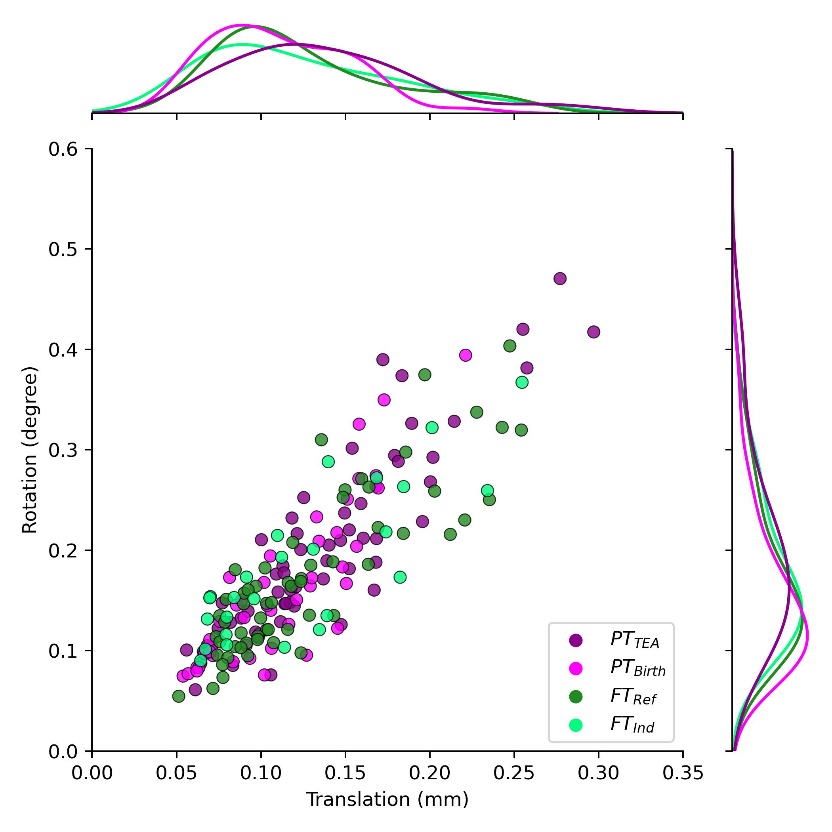


**Supp. Figure 2.** Quality control of diffusion data across the cohort showing estimated motion parameters (translation and rotation) for each infant. See Supp. Figure 1 legend for subject group legend. Importantly, diffusion data quality was visually comparable between preterm and full-term groups at both time points.


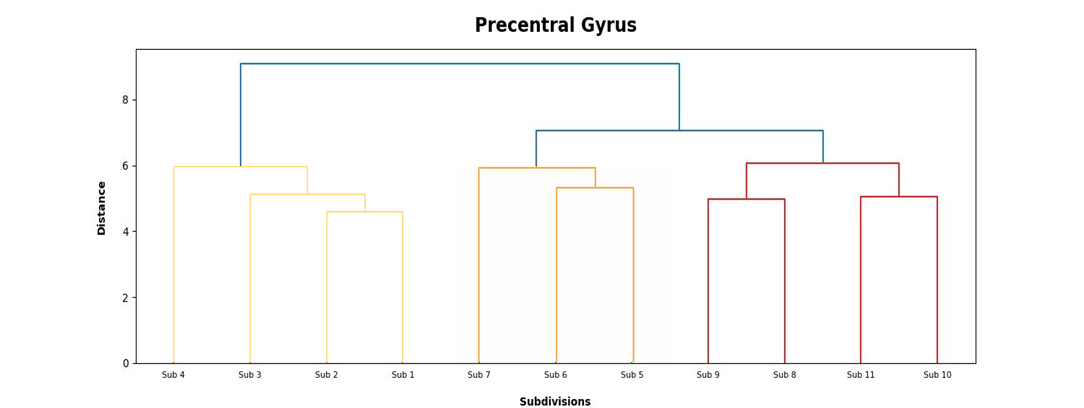


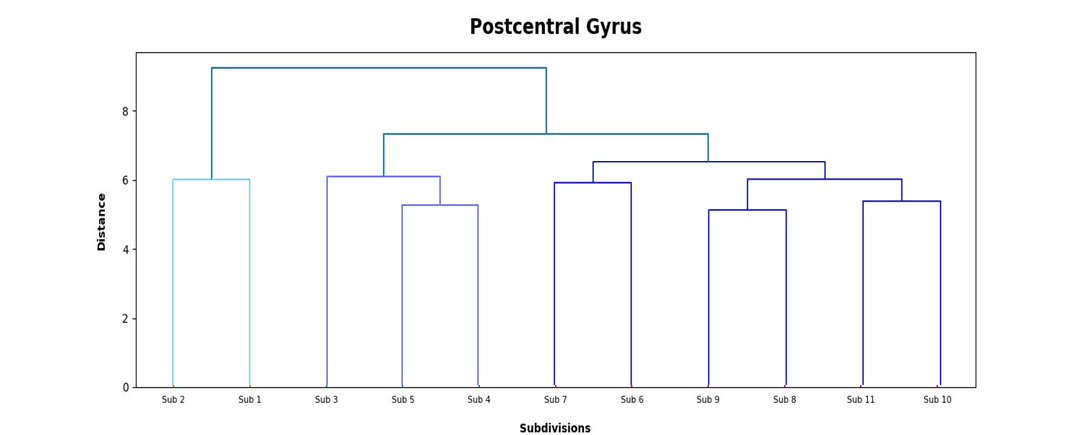


**Supp. Figure 3.** Dendrograms derived from agglomerative hierarchical clustering over the FT_Ind_ group (25 subjects). Each color represents an identified cluster in pre-central gyrus (top figure): Pre_LAT_, Pre_INT_, Pre_MED_ (yellow, orange, red, respectively); and post-central gyrus (bottom figure): Post_LAT_, Post_INT_, Post_MED_ (light blue, blue, indigo, respectively). See Figure 1 legend for cluster legend.

**Supp. Table 1.** Comparisons of FT_Ref_ and FT_Ind_ groups per cluster: Welch’s t-tests for each diffusion metric. Effect sizes are reported in terms of Cohen D. P-values are corrected for multiple comparisons across the 6 clusters (independently for each metric), and significant results are highlighted in green. See Supp. Figure 1 legend for subject group legend and Figure 1 legend for cluster legend. Note that degrees of freedom (dof) are non-integer and vary across comparisons due to the Welch correction for unequal variances used in the t-tests.

**FT_Ref_ vs. FT_Ind_**

|  | **AD** | | | **RD** | | | **MD** | | |
| --- | --- | --- | --- | --- | --- | --- | --- | --- | --- |
|  | **T (dof)** | **Cohen D** | **p_corr_** | **T (dof)** | **Cohen D** | **p_corr_** | **T (dof)** | **Cohen D** | **p_corr_** |
| **Pre_LAT_** | -1.78 (52.99) | 0.40 | 0.274 | -1.53 (56.59) | 0.33 | 0.341 | -1.72 (56.07) | 0.37 | 0.274 |
| **Pre_INT_** | -0.8 (50.94) | 0.18 | 0.570 | -0.53 (54.87) | 0.12 | 0.716 | -0.58 (53.65) | 0.13 | 0.698 |
| **Pre_MED_** | -1.2 (64.24) | 0.25 | 0.550 | -0.85 (56.65) | 0.19 | 0.570 | -0.86 (58.96) | 0.18 | 0.570 |
| **Post_LAT_** | -1.64 (48.88) | 0.38 | 0.298 | -1.97 (50.53) | 0.45 | 0.245 | -1.89 (50.83) | 0.43 | 0.257 |
| **Post_INT_** | -1.06 (42.74) | 0.26 | 0.550 | -0.99 (41.84) | 0.25 | 0.550 | -1.08 (41.54) | 0.27 | 0.550 |
| **Post_MED_** | -1.02 (48.33) | 0.24 | 0.550 | -0.94 (45.18) | 0.22 | 0.550 | -0.96 (46.6) | 0.23 | 0.550 |
|  | **FA** | | | **NDI** | | | **ODI** | | |
|  | **T (dof)** | **Cohen D** | **p_corr_** | **T (dof)** | **Cohen D** | **p_corr_** | **T (dof)** | **Cohen D** | **p_corr_** |
| **Pre_LAT_** | -0.33 (61.53) | 0.07 | 0.811 | 1.74 (56.21) | 0.38 | 0.274 | -2.29 (49.61) | 0.52 | 0.159 |
| **Pre_INT_** | -0.12 (53.87) | 0.03 | 0.934 | 0.45 (54.07) | 0.10 | 0.735 | 4.16 (58.21) | 0.90 | 0.001 |
| **Pre_MED_** | -0.19 (61.81) | 0.04 | 0.900 | 0.73 (56.43) | 0.16 | 0.603 | 5.02 (52.96) | 1.12 | <0.001 |
| **Post_LAT_** | 0.8 (50.96) | 0.18 | 0.570 | 2.13 (50.22) | 0.49 | 0.194 | -6.74 (55.61) | 1.47 | <0.001 |
| **Post_INT_** | 0.49 (60.6) | 0.10 | 0.731 | 1.11 (41.91) | 0.27 | 0.550 | -12.06 (54.55) | 2.66 | <0.001 |
| **Post_MED_** | 0.02 (54.78) | 0.00 | 0.988 | 0.97 (46.66) | 0.23 | 0.550 | -8.56 (80.08) | 1.58 | <0.001 |

**Supp. Table 2: Influence of group, region and position on dMRI metrics at term-equivalent age.**

**a,** Results of ANCOVA analysis examining the relationship between each diffusion metric and covariates, including PMA at scan, residual metric over whole cortex (corrected for PMA at scan), and factors of interest: group (FT_Ref_  / PT_TEA_), region (pre-/post-central gyrus), and position (lateral / intermediary / medial). The models account for hierarchical structure of the repeated measures across matched subjects, regions, and positions, with a nested error term. *Legend:* F: F-statistic from ANCOVA; p: statistical significance; partial η²: effect size; dof_1_/dof_2_: degrees of freedom of the effect/error. **b,** *Post-hoc* comparisons for the effect of regions in FT_Ref_ group (after concatenating values over positions within each region) using paired t-tests._._ **c,** *Post-hoc* comparisons for the effect of positions in FT_Ref_ group (after concatenating values over regions for each position) using paired t-tests. P-values were corrected for multiple comparisons across the 3 tests, independently for each metric (p_corr_). Significant results are highlighted in green. See Supp. Figure 1 legend for subject group legend and Figure 1 legend for cluster legend.

| **a, ANCOVA model** | **AD** | | | **RD** | | |
| --- | --- | --- | --- | --- | --- | --- |
|  | **F (dof_1_, dof_2_)** | **p** | **Partial η^2^** | **F (dof_1_, dof_2_)** | **p** | **Partial n2** |
| ***Covariates*** |  |  |  |  |  |  |
| Residual GM | - | - | - | - | - | - |
| PMA at scan | 107.33 (1,696) | <0.001 | 0.09708 | 88.37 (1,696) | <0.001 | 0.08387 |
| ***Factors*** |  |  |  |  |  |  |
| Group | 179.79 (1, 696) | <0.001 | 0.16261 | 173.11 (1,696) | <0.001 | 0.16431 |
| Region | 12.47 (1, 696) | <0.001 | 0.01128 | 11.44 (1,696) | 0.001 | 0.01085 |
| Position | 55.02 (2, 696) | <0.001 | 0.09953 | 42.34 (2,696) | <0.001 | 0.08037 |
|  | **MD** | | | **FA** | | |
|  | **F (dof_1_, dof_2_)** | **p** | **Partial η^2^** | **F (dof_1_, dof_2_)** | **p** | **Partial n2** |
| ***Covariates*** |  |  |  |  |  |  |
| Residual GM | - | - | - | 715.82 (1,695) | <0.001 | 0.47415 |
| PMA at scan | 95.67 (1,696) | <0.001 | 0.08875 | 2.07 (1,695) | 0.147 | 0.00137 |
| ***Factors*** |  |  |  |  |  |  |
| Group | 179.40 (1,696) | <0.001 | 0.16642 | 61.87 (1,695) | <0.001 | 0.04099 |
| Region | 13.11 (1,696) | <0.001 | 0.01216 | 4.87 (1,695) | 0.026 | 0.00323 |
| Position | 46.92 (2,696) | <0.001 | 0.08705 | 15.02 (2,695) | 0.000 | 0.01990 |
|  | **NDI** | | | **ODI** | | |
|  | **F (dof_1_, dof_2_)** | **p** | **Partial η^2^** | **F (dof_1_, dof_2_)** | **p** | **Partial n2** |
| ***Covariates*** |  |  |  |  |  |  |
| Residual GM | 2143.49 (1,695) | <0.001 | 0.50951 | 238.29 (1,695) | <0.001 | 0.18895 |
| PMA at scan | 293.10 (1,695) | <0.001 | 0.06967 | 136.19 (1,695) | <0.001 | 0.10798 |
| ***Factors*** |  |  |  |  |  |  |
| Group | 697.26 (1,695) | <0.001 | 0.16574 | 10.55 (1,695) | 0.001 | 0.00837 |
| Region | 46.73 (1,695) | <0.001 | 0.01111 | 77.74 (1,695) | <0.001 | 0.06164 |
| Position | 165.68 (2,695) | <0.001 | 0.07876 | 51.71 (2,695) | <0.001 | 0.08200 |

**b, Post-hoc tests on the effect of region**

**FT_Ref_ :** pre- vs post-central gyrus

|  | **T (dof)** | **Cohen D** | **p** |
| --- | --- | --- | --- |
| **AD** | -6.74 (176) | 0.24 | <0.001 |
| **RD** | -7.55 (176) | 0.28 | <0.001 |
| **MD** | -7.9 (176) | 0.29 | <0.001 |
| **FA** | 3.22 (176) | 0.22 | 0.002 |
| **NDI** | 6.58 (176) | 0.25 | <0.001 |
| **ODI** | 7.76 (176) | 0.55 | <0.001 |

**c, Post-hoc tests on the effect of position**

**FT_Ref_ :** LAT vs INT vs MED

|  | **LAT vs INT** | | | **LAT vs MED** | | | **INT vs MED** | | |
| --- | --- | --- | --- | --- | --- | --- | --- | --- | --- |
|  | **T (dof)** | **Cohen D** | **p_corr_** | **T (dof)** | **Cohen D** | **p_corr_** | **T (dof)** | **Cohen D** | **p_corr_** |
| **AD** | 22.16 (117) | 1.12 | <0.001 | 24.32 (117) | 1.61 | <0.001 | 12.76 (11 | 0.61 | <0.001 |
| **RD** | 13.42 (117) | 0.64 | <0.001 | 23.59 (117) | 1.48 | <0.001 | 18.8 (117) | 0.90 | <0.001 |
| **MD** | 17.25 (117) | 0.82 | <0.001 | 24.08 (117) | 1.54 | <0.001 | 17.45 (117) | 0.80 | <0.001 |
| **FA** | 7.91 (117) | 0.62 | <0.001 | -2.91 (117) | 0.29 | 0.004 | -9.26 (117) | 0.85 | <0.001 |
| **NDI** | -15.93 (117) | 0.76 | <0.001 | -23.21 (117) | 1.48 | <0.001 | -16.73 (117) | 0.79 | <0.001 |
| **ODI** | -8.89 (117) | 0.82 | <0.001 | -13.95 (117) | 1.26 | <0.001 | -4.44 (117) | 0.37 | <0.001 |

**Supp. Table 3**. Assessment of the nullity of Mahalanobis distances in PT_Birth_ and PT_TEA_ groups: t-tests for each cluster are compared to 0 (i.e. mean of the reference group). P-values are corrected for multiple comparisons across the 6 clusters for each group independently. See Supp. Figure 1 legend for subject group legend and Figure 1 legend for cluster legend.

|  | **PT_Birth_** | | | **PT_TEA_** | | |
| --- | --- | --- | --- | --- | --- | --- |
|  | **T (dof)** | **Cohen D** | **p_corr_** | **T (dof)** | **Cohen D** | **p_corr_** |
| **Pre_LAT_** | 38.516 (44) | 5.742 | <0.001 | 24.876 (58) | 3.239 | <0.001 |
| **Pre_INT_** | 45.321 (44) | 6.756 | <0.001 | 26.929 (58) | 3.506 | <0.001 |
| **Pre_MED_** | 41.567 (44) | 6.196 | <0.001 | 19.733 (58) | 2.569 | <0.001 |
| **Post_LAT_** | 32.000 (44) | 4.770 | <0.001 | 23.703 (58) | 3.086 | <0.001 |
| **Post_INT_** | 40.994 (44) | 6.111 | <0.001 | 27.102 (58) | 3.528 | <0.001 |
| **Post_MED_** | 40.994 (44) | 5.999 | <0.001 | 22.937 (58) | 2.986 | <0.001 |
